# Chicago Medical Society, May Meeting

**Published:** 1884-09

**Authors:** 


### SOCIETY PROCEEDINGS.

---

CHICAGO MEDICAL SOCIETY. *Regular Meeting, 19th May, 1884. The President,* DR. D. A. K. STEELE *in the chair.*—MONOLOCULAR OVARIAN CYST, *exhibited by* PROFESSOR CHAS. T. PARKES, M. D.—UTERINE LEIO-MYOMATA, *exhibited by* PROFESSOR E. C. DUDLEY, M. D.—REPORT OF COMMITTEE ON STATE MEDICINE.

Professor Chas. T. Parkes, M. D. gave the clinical history of a monolocular ovarian cyst, weighing twenty-four pounds. The cyst was exhibited. The patient, nineteen years old, had noticed during the last three years a gradually increasing, abdominal, tumor. She had been treated by a number of physicians for dropsy. Three weeks ago, she came under Dr. Parkes's observation, and the diagnosis of monolocular cyst was made. The tumor increased in size until the abdominal circumference, measured around the umbilicus, was forty-four inches. She enjoyed excellent health, and experienced no especial inconvenience from the tumor.

Four days since, in one of the city hospitals, the cyst was removed.

An incision in the median line of the abdomen,—three and one-half inches in length,—was made, the cyst tapped, twentythree pounds of fluid removed, and the characteristic, broad pedicle ligatured.

There were no intestinal or omental adhesions. The sac of the cyst weighed one pound, and, when distended with air showed no corrugations. The hæmorrhage was trifling, and was controlled by ligatures. Since the operation, the patient's temperature has not risen above 99 1-5° F., and the pulse has not exceeded 100.

An uneventful convalescence is anticipated.

Professor E. C. Dudley, M. D. exhibited specimens of two leio-myomata, removed from the uterus by laparotomy. The following is the substance of his verbal report of the case, which presents few parallels in the history of laparotomy.

The patient was referred to Dr. Dudley by Professor R. N. Isham, M. D.

The patient, forty-eight years old, Norwegian, never married, never pregnant, cessation of menstrual epoch last fall; the tumor had developed in two years.

The patient was seen for the first time, by the operator, last January. The abdomen was enormously distended,—at least three times as large as in pregnancy at term,—by a solid mass, which on aspiration yielded negative results. On percussion, *tympanites*, in the right flank and upper epigastric region, was elicited, while flatness was found in the other abdominal regions.

The uterus had been displaced upwards so far as to destroy all outline of the vaginal portion of the cervix. With great difficulty, the *os externum* could be reached by the tip of the finger. It was in extreme ante-location on a level with the pelvic brim. The *corpus* could not be outlined. The posterior *cul de sac* was filled with a mass, which crowded it down to a level with the perineum. The introduction of the sound was impossible. *Per rectum*, the mass appeared non-adherent. A sound in the bladder passed nearly to the umbilicus. The patient was greatly emaciated.

The operator's diagnosis, at this time, was solid tumor of the uterus, with such extensive attachments, that removal would be almost certainly fatal, and exploratory incision was not to be considered, because the extreme distension of the abdominal walls would have prevented closing the wound over the tumor. The operator's advice to the patient,—like that of a number of eminent surgeons, who had previously seen her,—was adverse to operation. The patient returned after a few weeks, extremely emaciated, suffering from dyspnœa, gastric disturbance, and begged for the operation, as a *dernier ressort*.

The treatment, immediately preparatory to the operation consisted of two Turkish baths, thorough antiseptic irrigation of the vagina, and moderate catharsis.

All furniture in the patient's room, and that immediately adjoining it,—not absolutely necessary,—was removed. Ceiling, walls and wood-work were antiseptically cleansed. The rooms were thoroughly fumigated with burning sulphur for twenty-four hours. The *mons veneris* was shaved; the abdomen and genital canal were thoroughly cleansed by repeated applications of corrosive sublimate.

The instruments were passed through the flame of a spirit lamp, wiped by means of a bit of cotton, with pure carbolic acid, and immersed in a dilute solution of corrosive sublimate, (1 : 4000). The ligatures,—silk,—were first boiled in 95 per cent. sol. of carbolic acid for ten minutes, then in a 5 per cent. sol. for half an hour. Equally scrupulous, detailed attention was given to the disinfection of the assistants. In short, the conditions were as nearly as possible those of absolute antisepsis.

The operation was performed 17th March, 1884.

Etherization was effected by Dr. R. Tilley with Ormsby's inhaler.

The operator was assisted by Drs. R. N. Isham, C. Fenger, W. W. Jaggard, W. P. Verity, George Isham, and O. E. Larkin.

The abdominal incision was made from umbilicus nearly to the ensiform appendix; it was prolonged downwards about two inches to the bladder, which had been drawn out of place.

If the incision had been made in the usual manner, the bladder would have been penetrated,—as it was found universally adherent over the anterior surface of the tumor. The transverse colon, pushed by the tumor in its growth, was found firmly adherent for a distance of eight inches in the upper epigastric region. A bundle of vessels, one inch in diameter, penetrated the tumor, just below the adherent colon. A nearly spherical, solid tumor, fourteen inches in diameter, springing from the left posterior wall of the *corpus uteri*, developing between the folds of the broad ligament,—which enveloped not less than half of its surface on the left and posteriorly,—with its anterior and inferior surfaces covered by the adherent bladder, presented itself. The tumor was also extensively attached posteriorly to the peritoneal surfaces. To the right of the tumor, lay the intestines, unattached.

The removal of the tumor *seemed* impossible, retreat *was* impossible. The adhesions and attachments were broken up by tearing, dissection and enucleation. All bleeding vessels were so carefully ligatured, as soon as wounded, that when the tumor was lifted out, the abdominal cavity was dry. In dissecting off the attached bladder, the *urachus* was opened. This gave rise to a spirt of urine. The opening was secured by a ligature. The tumor was now freed from all except its uterine attachments, which embraced the entire left posterior portion of the *corpus*. Around this attachment,—too short to be designated as a pedicle,—was passed, four times, a solid, round, pure rubber cord, ¼ inch in diameter, and securely tied. Hemorrhage, which, during the long and tedious enucleation, had, at times, been severe, was now well controlled. The tumor was then cut away, by an incision about three inches on the distal side of the rubber ligature. This left a pedicle, composed of uterine tissue, five inches in diameter. Another tumor, filling the *pelvis minor*, of the size and shape of the fœtal skull, springing from the posterior wall of the uterus, by an attachment two inches in diameter, was ligatured and removed in the same way. These ligatures had been placed temporarily with the expectation of removing them and of substituting clamps and the extra-peritoneal treatment; but now, after nearly two hours of rapid operating,—in which the peritoneal surfaces were extensively injured,—the patient showed unmistakable signs of impending collapse. The danger of substituting the clamp for the temporary ligatures was too great, so the latter were made permanent, and the intra-peritoneal plan adopted. Perforated, soft rubber drainage tubes, two-thirds of an inch in diameter, were placed respectively in the anterior and posterior *cul de sac*. The abdominal incision,—which, during the operation, had been lengthened to eighteen inches,—was closed with silk sutures, and dressed antiseptically. Immediately after the operation, the patient's pulse was scarcely perceptible, but she rallied from shock with surprising rapidity. Temperature, during the first eight days, ranged from 98–101° F.; pulse, 100 to 120; respiration was accelerated to a corresponding degree; flatus passed downwards on the second day. On the third day the drainage tubes were removed, no discharge having escaped through them. No unfavorable symptoms were observed until the ninth day, when the temperature, in the morning, rose to 102° F., in the evening, to 103° F.; pulse was correspondingly accelerated; during the eleventh and twelfth days, temperature reached 104–5° F., pulse and respiration keeping equal pace.

Professor C. Fenger, M. D. was summoned to a consultation. A rapidly increasing tumor, observed in the lower hypogastric region, rising out of the pelvis, of a non-fluctuating character, was thought to be pus confined within tense walls.

An abdominal incision was made and about one pint of thick, fœtid pus was discharged from a cavity, which nature had made by the agglutination of peritoneal surfaces around it.

This cavity was washed out and soft rubber drainage tubes re-introduced to the bottom of Douglas's *cul de sac*, which bounded the cavity inferiorly, where the end of the tube could be easily felt by the vaginal touch. The abscess cavity was frequently washed out with dilute solutions of carbolic acid.

For two days, temperature and pulse continued nearly normal; the temperature, finally, again rose to 104–5° F. Examination revealed no new accumulation of pus, and the rise in temperature being attributed to imperfect drainage, a counter-opening was made, between the *cul de sac* of Douglas and the vagina, about the twelfth day, and the drainage tube was passed downwards to the vulva. This opening was preceded by careful disinfection of vagina and vulva. An antiseptic dressing of cotton and iodoform was placed over the vulva.

From this time, temperature and pulse gave little anxiety. The patient rapidly improved. All bodily functions became nearly normal. The patient's appetite was voracious. Surprising quantities of food were consumed. The cavity continued to discharge pus, and was washed out with greater or less frequency according to the temperature. On the twenty-seventh day, introducing the finger into the cavity through the abdominal wound, the lesser ligature was hooked out, together with some adherent silk thread. On the thirty-seventh day, the larger ligature, with a large sloughing mass, was, in the same way, removed. On the forty-first day, the drainage tube was drawn up into the cavity and the opening through the *cul de sac* closed spontaneously in a few days, so as to leave but one opening,—that is, through the lower angle of the abdominal incision.

Now, on the sixty-third day after the operation, the patient's convalescence may be regarded as secure. Pallor has left her face, she has greatly increased in flesh, sleeps perfectly, and, were it not for the slight drainage, would consider herself well.

The larger tumor, after the escape of much fluid, weighed thirty pounds; the smaller tumor weighed two pounds. Professor Lester Curtis, M. D., on microscopic examination, pronounced the tumors to be *leio-myomata*.

The case illustrated a few truths.

It establishes a wide latitude of possibilities for operation in the abdominal and pelvic cavities. It almost implies that we may be on the eve of operating in the most dangerous cases of uterine myomata quite as safely as in the more serious cases of ovarian cyst. The operator discussed at length the subjects of elastic and inelastic ligatures, intra and extra-peritoneal plans of treatment in laparo-myomotomy, and expressed the hope that future experience would establish the laws governing the intraperitoneal treatment of the pedicle upon as sound a basis as in the case of ovarian cysts. L. H. M.

